# Effects of Bright Light with Reduced Blue Light on Sleepiness on Rising: A Small Exploratory Study

**DOI:** 10.1155/2018/2378630

**Published:** 2018-10-04

**Authors:** Jun Miura, Tomonori Yuasa, Yasunori Sugai, Kana Yamagami, Yoshihisa Aizu

**Affiliations:** ^1^Faculty of Pharmaceutical Sciences, Hokkaido University of Science, Sapporo 006-8585, Japan; ^2^Health Administration Center, Muroran Institute of Technology, Muroran 050-8585, Japan; ^3^College of Design and Manufacturing Technology, Muroran Institute of Technology, Muroran 050-8585, Japan; ^4^Densei Communication Inc., Ebetsu 067-0051, Japan

## Abstract

Bright light therapy is a treatment modality for seasonal affective disorder and circadian rhythm disorders in which artificial light of 2,500 lux or higher at the eye is effective. Although short-wavelength visible light is more effective than long-wavelength visible light, it may be hazardous to the retina. Recently, light-emitting diodes (LEDs) have been used as the light source in bright light therapy apparatuses. We developed goggles for bright light therapy equipped with LEDs as the light source. The aim of this study was to examine the efficacy and safety of our goggles when emitting 10,000-lux light with its short-wavelength light content reduced by 30% or 50% (denoted as 30%-cut and 50%-cut light, respectively, henceforth). Six healthy young males participated in this study. They were administered no light, 50%-cut light, and 30%-cut light for 30 min early in the morning for 4 days each. Subjective sleepiness and sleep quality were evaluated by the Stanford Sleepiness Scale (SSS) and the Oguri–Shirakawa–Azumi sleep inventory MA version (OSA-MA), respectively. Subjective sleepiness evaluated by the SSS and the subscale of the OSA-MA significantly decreased with 30%-cut light compared with no light. Psychomotor performance evaluated by a calculation task improved with the 30%-cut light, although not significant after multiple comparisons were considered. No abnormality was found by ophthalmoscopy and the vision test. In conclusion, our goggles with 30%-cut light may be safe and have an awakening effect.

## 1. Introduction

Bright light therapy is a treatment modality in which artificial light of 2,500 lux or higher at the eye is administered to patients. It is effective for conditions such as seasonal affective disorder (SAD) [1, 2] and circadian rhythm sleep disorders [3]. In a previous study on SAD, 2,500-lux fluorescent light was administered for 2 h [1], but a subsequent study showed that 10,000-lux light for 30 min resulted in similar efficacy [4]. This has therefore become the treatment protocol used in clinical settings.

Several physiological and biochemical effects of bright light have been reported. First, bright light suppresses nocturnal melatonin secretion from the pineal body [5, 6]. Second, sleepiness is reduced and alertness is improved, possibly owing to the suppression of melatonin [7]. Third, bright light improves psychomotor performance. For example, daytime exposure to bright light has improved performance in a psychomotor vigilance task [8]. Fourth, bright light shifts the circadian rhythm; i.e., it advances and delays the rhythm when it is administered in the morning and evening, respectively [2, 4].

The spectral characteristics of the light source for bright light therapy apparatus have been of great concern. Short-wavelength visible light with a peak of 460 nm (blue light) is more potent in suppressing melatonin secretion than is long-wavelength visible light with a peak of 555 nm [5]. In clinical applications, longer-wavelength light is relatively ineffective for SAD whereas light of short and medium wavelengths has produced antidepressant effects [9]. However, exposure to short-wavelength light may be hazardous; it has been shown to cause retinal damage in rats [10]. Recently, light-emitting diodes (LEDs) have been replacing fluorescent lamps as a light source not only in room lamps, but also in bright light therapy apparatus. Since LED products emit an amount of short-wavelength light, it is recommended that a film is attached to reduce short-wavelength light and protect the retina [11].

**Figure 1:**
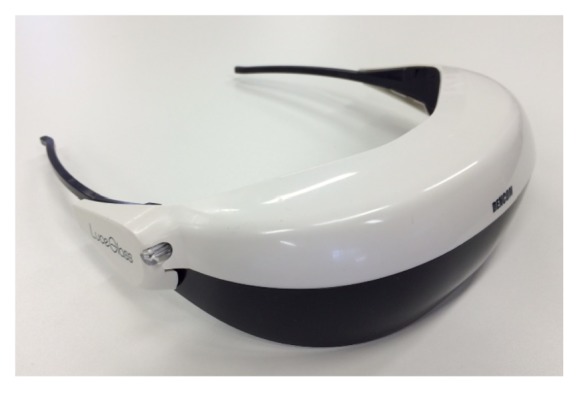
Goggles for bright light therapy. LEDs at the back of the frame are used as the light source.

We have developed portable goggles for bright light therapy (Figure 1) using LEDs as the light source [12]. To minimize the possible hazard to the retina, we have reduced the emission of short-wavelength light by 30% or 50% while keeping an intensity of 10,000 lux at the cornea. This study, therefore, aims to investigate the efficacy and safety of our goggles with different content of short-wavelength light. Thus, the primary endpoint of this study was subjective sleepiness after light irradiation. The secondary endpoints were psychomotor performance and possible hazard to the retina.

## 2. Materials and Methods

### 2.1. Subjects

The clinical trial enrolled six male volunteers from among students of Muroran Institute of Technology. All were healthy and aged 22 or 23 years. They were not on medication, had no history of serious illness, or had no complaint of sleep disturbance. The subjects underwent ophthalmoscopy and vision tests performed by an ophthalmologist in Muroran prior to commencing the study, and no abnormality was found. All subjects signed an informed consent form after the purpose and design of this study were explained. The study was approved by the Ethics Committee of the Muroran Institute of Technology, registered in the clinical trial database (UMIN000025694), and carried out in accordance with the ethical principles of the Declaration of Helsinki.

### 2.2. Phototherapy Goggles

**Figure 2:**
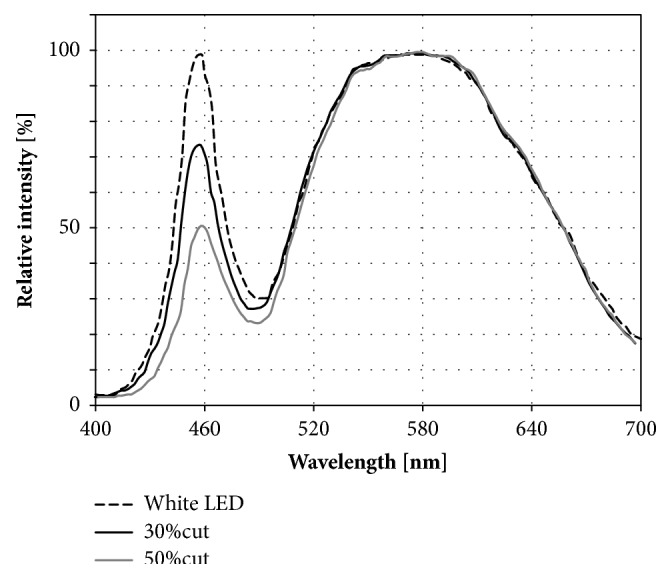
The relative spectral radiant intensity of light with short-wavelength content reduced by 30% or 50% compared to white LED.

The phototherapy goggles (Figure 1) were prepared by the authors [12]. LEDs (Cree, Durham, NC) were fitted to the back of the frame. A light yellow band-stop filter (Tuftop®, Toray, Tokyo, Japan) was attached on the light diffusion board to reduce the content of short-wavelength light by 50% or 30% (denoted as 50%-cut or 30%-cut light, respectively). The spectral radiant intensity of the light compared with white LED was shown in Figure 2. Light was irradiated onto the eye at an angle of 55° with an intensity of 10,000 lux at the cornea. The hazard analysis showed that the permitted exposure time of 30%-cut light was 79 min in accordance with safety standard JIS C7550 [12].

### 2.3. Questionnaires

Subjective sleepiness was evaluated using the Stanford Sleepiness Scale (SSS). This is a single scale scored from 1 to 7, with a higher score indicating a greater feeling of sleepiness. Subjective sleep quality was evaluated using the Oguri–Shirakawa–Azumi sleep inventory MA version (OSA-MA) [13]. This is a self-report questionnaire composed of 16 items each with a 4-point scale. The items are consolidated into five subscales: factor I “sleepiness on rising,” factor II “initiation and maintenance of sleep,” factor III “frequent dreaming,” factor IV “refreshing,” and factor V “sleep length.” The Zi value was calculated using an MS-Excel sheet provided by the creators of the inventory, with higher values indicating better sleep quality.

### 2.4. Psychomotor Performance

**Figure 3:**
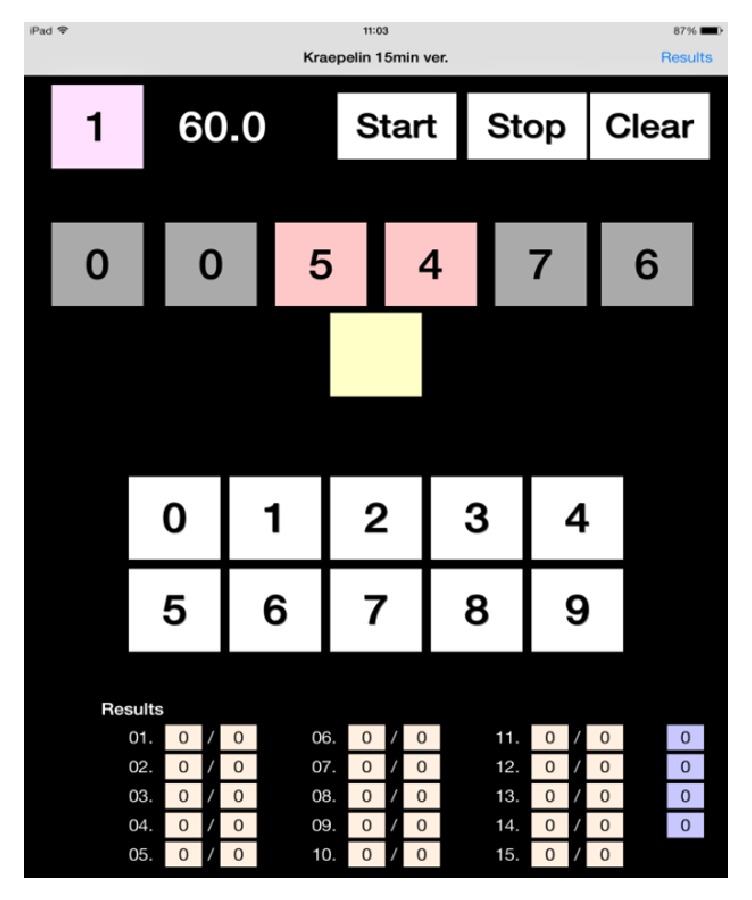
The calculation task application working on an iPad mini®.

To facilitate a straightforward evaluation of psychomotor performance, we have developed a simple calculation task application that runs on an iPad mini® [14]. Briefly, in each trial, six digits were shown on the screen (Figure 3), and the subject was instructed to add together the middle two figures and then to tap the numbered square below which corresponded with the result, or with the right-hand digit of the result if it exceeded 10. The task lasted for 5 min and the numbers of trials completed and correct answers were automatically calculated. A film filtering blue light was attached to the screen of the iPad mini®. We have previously reported that the number of correct answers was significantly lower immediately after getting up in the morning than in the daytime and in the evening, and that the number of correct answers significantly increased after dawn simulation [14]. Thus, this calculation task was used to evaluate psychomotor performances.

In the preliminary study, the training effect of the calculation task was examined by administering it 20 times in daytime to nine college students of the Hokkaido University of Science. The ceiling effect was statistically examined by the Shirley-Williams test.

### 2.5. Experimental Design

The clinical trial consisted of three courses of intervention. In the first course, the goggles were used without the LEDs (“no light”). In the second and third courses, the goggles with 50%-cut light and those with 30%-cut light were used, respectively. Each course lasted for 4 days, with a 4-day interval between the courses. The subjects refrained from working after 2200 and drinking alcohol throughout the whole study period. From 2200 on the night before the intervention, the subjects stayed under dim light in an air-conditioned, temperature-controlled room (at 20°C) with shade curtains. During the intervention courses, the subjects were woken up at 0450, which was around 2 h earlier than usual. The subjects then wore the goggles from 0500 to 0530 under dim light. They were instructed to open their eyes sufficiently to let in the light, but without gazing at the light source. Subsequently the subjects performed the calculation task for 5 min. Finally, subjective sleepiness and sleep quality were evaluated by the questionnaires. The research assistant attended the intervention to ensure the compliance. After this, the room light was turned on and the subjects were allowed to spend time as they liked. During the intervals between courses, neither in-bed nor out-of-bed time was controlled. In order to exclude training effects, subjects were instructed to practice the calculation task at least ten times before the study started.

### 2.6. Statistical Analysis

The Friedman test was used to assess differences across the study for the number of correct answers in the calculation task, the SSS score, and the scores for OSA-MA factors I to V. If statistically significant, the results for day 2 – 12 were compared with the result for day 1 by the Wilcoxon Signed Rank test. In addition, the results for no light, 50%-cut light and for 30%-cut light were compared by the Kruskal Wallis test followed by the Steel-Dwass test. The statistical analyses were carried out by Bell Curve Excel statistics for Windows (SSRI, Tokyo, Japan), and a P value <0.05 was considered statistically significant.

## 3. Results 

The SSS score did not differ significantly across the study (P=0.060) (Table 1). However, the scores for the three interventions were significantly different (P=0.003). The Steel-Dwass test revealed that the score for 30%-cut light was significantly lower than that for no light (P=0.002), indicating that the subjects were less sleepy during the course.

Concerning the OSA-MA scales, the score for the factor I did not differ significantly across the study (P=0.061) (Table 2). However, the scores for the three interventions were significantly different (P=0.004). The Steel-Dwass test revealed that the score for 30%-cut light (48.2 ± 8.7) was significantly higher than that for no light (39.1 ± 7.5) (P=0.003). Since the factor I referred to sleepiness on rising, the subjects might have been less sleepy during the period. In contrast, the Friedman test did not show any change in the scores for the factors II, III, IV, and V across the study (P=0.304, 0.957, 0.643, and 0.242, respectively).

**Figure 4:**
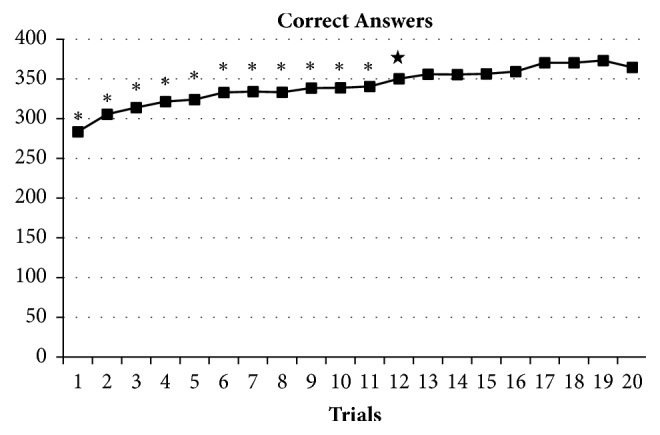
Mean number of correct answers in the calculation task up to the trial 20.  ^*∗*^P < 0.05 versus the trial 20, ^★^P < 0.05 versus the mean of trials 16 – 20.

Figure 4 shows mean number of correct answers in the calculation task up to the trial 20 in the preliminary study. The Shirley-Williams test shows that the number of correct answers for the trial 20 was significantly higher than that for the trials 1 to 11 (P < 0.05). In addition, the mean number of correct answers for the trials 16 to 20 was significantly higher than that for the trials 1 to 12 (P < 0.05).

In the clinical trial, the number of correct answers in the calculation task significantly differed across the study (P=0.003) (Table 3). Compared with day 1, the number of correct answers increased on day 8 (P=0.028), day 9 (P=0.028), day 11 (P=0.028), and day 12 (P=0.043). These interday variations, however, did not reach the significant level after correction by the number of comparizons, i.e., Bonferroni method. The number of correct answers in the calculation task tended to differ across the intervention (P=0.072). The number of correct answers for 30%-cut light tended to increase compared with that for no light (P=0.085).

After the study was completed, the ophthalmoscopy and vision tests were repeated by the same ophthalmologist. Again, no abnormality was found.

## 4. Discussion

This study explored the efficacy and safety of bright LED light (at 10,000 lux) with its short-wavelength content reduced by 30% or 50%. This was tested in a situation in which the subjects had to wake up earlier than usual. Both the score for SSS and the OSA-MA score for factor I indicated less sleepiness on rising with 30%-cut light than with no light. These results indicate that our goggles equipped with 30%-cut light may have a subjective awakening effect. Our goggles with 30%-cut light may therefore be a good tool to alleviate sleepiness in situations in which people feel sleepy, such as with jet-lag [8], shift working [3], seasonal affective disorder [1], or circadian rhythm sleep disorders [3].

In contrast to SSS and the OSA-MA score for factor I, the scores for the OSA-MA factors II to V did not change significantly with the intervention. This suggests that our goggles may not affect subjective sleep quality. This may be due to the fact that the subjects were healthy and did not suffer from sleep disturbance.

The number of correct answers in the calculation task significantly differed across the study, but post hoc comparisons with day 1 did not reach the significant level after multiple comparisons were taken into consideration. The discrepancy between subjective sleepiness and psychomotor performance may be due to the small sample size and/or the small effect size on the calculation task. From the result, at least 30 participants will be needed to obtain statistical significance when assuming mean ± S.D. number of correct answers to be 376 ± 35 and 401 ± 53 by no light and 30%-cut light, respectively.

In discussing awakening effects of our goggles, circadian entrainment and/or sleep deprivation should be considered since the subjects were woken up around 2 h earlier than usual during the intervention. Our experimental design mimicked the situation in which people flew eastward and had to advance the circadian clock. An early report has suggested that the circadian clock phase advanced 57 min/day after eastward flights [15]. According to this theory, the subjects of this study should have reentrained the circadian clock in the first few days, namely in the no light period. Nevertheless, subjective sleepiness improved in the 30%-cut light period (i.e., from day 9). Therefore, the reduction in sleepiness may be due to light irradiation rather than circadian reentrainment.

Concerning the safety measurement in the clinical trial, no abnormalities were found in the subjects' retinas or eyesight after the study. Taken together with the previous hazard analysis [12], our goggles with 30%-cut light may be safe in so far as they are used properly for up to 4 days. However, as bright light therapy may last longer than 4 days, long-term safety evaluation tests are desirable.

Limitations of this study include the limited number and sex of the subjects, and the study protocol. Since this was a small exploratory study, only six young men were recruited to exclude the effects of menstruation. Another drawback of this study was the sequence of the intervention; all the participants were administered no light, 50%-cut light, and 30%-cut light in the first, second and third courses, respectively, with a 4-day interval between the courses. Since the amount of short-wavelength content administered increased along with the courses, the additive effect should be concerned.

## 5. Conclusions

In conclusion, our goggles equipped with the 30%-cut light appear to be safe and have a subjective awakening effect. To verify our findings, larger scale studies including female subjects with a different experimental design such as a cross-over design are desirable.

## Figures and Tables

**Table 1:** Effects of the interventions on the Stanford Sleepiness Scale score. Data are presented as mean ± S.D.

Intervention	Day	Stanford Sleepiness Scale (n=6)	Mean Stanford Sleepiness Scale	P vs No light
No light	1	3.0 ± 1.0	3.9 ± 1.4	−
	2	3.7 ± 1.4		
	3	4.5 ± 1.4		
	4	4.3 ± 1.5		

50%-cut	5	3.2 ± 1.0	3.3 ± 1.5	0.148
	6	3.3 ± 2.0		
	7	3.2 ± 1.6		
	8	2.5 ± 0.8		

30%-cut	9	2.2 ± 0.8	2.6 ± 1.1	0.002
	10	2.8 ± 1.5		
	11	2.5 ± 0.8		
	12	2.8 ± 1.2		

**Table 2:** Effects of the interventions on the mean scores for Oguri–Shirakawa–Azumi sleep inventory factors I to V. Factor I is “sleepiness on rising,” factor II “initiation and maintenance of sleep,” factor III “frequent dreaming,” factor IV “refreshing,” and factor V “sleep length.” Data are presented as mean ± S.D.

Intervention	Day	Factor I (n=6)	Mean Factor I	P vs No light

No light	1	43.8 ± 5.4	39.1 ± 7.5	−
	2	38.2 ± 8.1		
	3	37.6 ± 8.3		
	4	36.6 ± 7.7		

50%-cut	5	42.1 ± 9.2	43.1 ± 9.9	0.209
	6	42.5 ± 11.5		
	7	43.0 ± 7.7		
	8	45.0 ± 12.9		

30%-cut	9	51.6 ± 8.7	48.2 ± 8.7	0.003
	10	46.6 ± 10.3		
	11	48.0 ± 8.5		
	12	46.7 ± 8.5		

Intervention	Day	Factor II (n=6)	Mean Factor II	

No light	1	41.7 ± 10.7	40.8 ± 9.8	
	2	39.2 ± 6.1		
	3	44.2 ± 13.3		
	4	38.1 ± 9.0		

50%-cut	5	40.7 ± 5.6	40.5 ± 8.2	
	6	34.1 ± 7.0		
	7	42.7 ± 8.6		
	8	44.5 ± 9.1		

30%-cut	9	40.0 ± 10.2	42.8 ± 10.1	
	10	47.7 ± 9.6		
	11	44.2 ± 11.1		
	12	39.2 ± 9.9		

Intervention	Day	Factor III (n=6)	Mean Factor III	

No light	1	50.4 ± 12.6	49.2 ±11.7	
	2	50.4 ± 12.6		
	3	51.5 ± 11.6		
	4	44.3 ± 11.8		

50%-cut	5	54.9 ± 8.2	53.8 ± 8.8	
	6	53.4 ± 12.2		
	7	56.2 ± 5.5		
	8	50.7 ± 9.3		

30%-cut	9	53.4 ± 8.4	51.3 ± 11.6	
	10	53.2 ± 9.3		
	11	49.3 ± 14.8		
	12	49.3 ± 14.8		

Intervention	Day	Factor IV (n=6)	Mean Factor IV	

No light	1	42.8 ± 8.4	41.6 ± 7.6	
	2	41.5 ± 6.2		
	3	40.1 ± 9.7		
	4	38.8 ± 8.6		

50%-cut	5	43.3 ± 10.0	42.5 ± 7.7	
	6	33.7 ± 7.4		
	7	45.2 ± 6.2		
	8	40.1 ± 11.2		

30%-cut	9	44.1 ± 6.0	45.1 ± 6.7	
	10	40.1 ± 6.3		
	11	48.1 ± 8.1		
	12	44.1 ± 8.1		

Intervention	Day	Factor V (n=6)	Mean Factor V	

No light	1	42.8 ± 8.4	40.8 ± 7.9	
	2	38.8 ± 8.6		
	3	45.2 ± 6.2		
	4	40.1 ± 6.3		

50%-cut	5	41.5 ± 6.2	40.6 ± 9.4	
	6	43.3 ± 10.0		
	7	40.1 ± 11.2		
	8	48.1 ± 8.1		

30%-cut	9	40.1 ± 9.7	44.1 ± 7.3	
	10	33.7 ± 7.4		
	11	44.1 ± 6.0		
	12	44.1 ± 8.1		

**Table 3:** Effects of the interventions on the number of correct answers in the calculation task across the study. Data are presented as mean ± S.D.

Intervention	Day	Correct answers (n=6)	P vs Day 1	Mean correct answers	P vs No light
No light	1	377 ± 51	−	376 ± 35	−
	2	380 ± 36	0.684		
	3	375 ± 32	0.833		
	4	368 ± 24	0.917		

50%-cut	5	375 ± 47	0.528	384 ± 47	0.859
	6	381 ± 26	0.463		
	7	383 ± 54	0.399		
	8	397 ± 63	0.028		

30%-cut	9	394 ± 55	0.028	401 ± 53	0.085
	10	396 ± 51	0.058		
	11	409 ± 63	0.028		
	12	403 ± 55	0.043		

## Data Availability

The statistical data used to support the findings of this study are included within the article. The detailed data are available from the corresponding author upon request.
